# Food as Software: Place, Protein, and Feeding the World Silicon Valley–Style

**DOI:** 10.1080/00130095.2020.1834382

**Published:** 2020-11-18

**Authors:** Alexandra E. Sexton

**Affiliations:** Oxford Martin School University of Oxford Oxford, OX1 3BD UK and Department of Geography University of Sheffield Sheffield, S3 7ND UK

**Keywords:** food, place, Silicon Valley, culture, innovation

## Abstract

This article examines the role of place—specifically the place of Silicon Valley in California—in the emerging economic geographies of alternative proteins (APs), including cellular and plant-based substitutes. Drawing on original fieldwork data and existing economic geography debates on food, place, and innovation, I develop the concept of *innovation terroir* to examine the key role Silicon Valley has played in shaping the spatial trajectories and political possibilities of the AP sector. I first illustrate the power of Silicon Valley’s place-myth in (re)producing the importance for AP founders to be *physically* in place within the region, in part to access its renowned industrial resources but also to provide a protective niche of credibility and credulity for these nascent ventures. Second, I outline that to be there in spatial terms has also involved an encountering with a specific culture of logics and practices of the tech-noindustrial region of Silicon Valley. To succeed at doing protein food in this region has required a choice by AP ventures to become *culturally* in place and thereby reimagine food through the Valley’s image of high-tech entrepreneurial innovation; in short, it has required food to become software. This exploration of cultural emplacement builds directly on recent work in geography and related fields on alternative food economies, geographies of innovation, and the ontological politics of APs. It offers timely contributions for considering how AP development might be done otherwise and what it means to look to Silicon Valley for solutions to global food security and broader planetary challenges.

*With the latest tech, U.N. seeks to end hunger Silicon Valley–style*. (Mis 2016)

In this article I consider the role of place—specifically the place of Silicon Valley, California—in the emerging economic geographies of alternative proteins (APs). The two AP approaches I explore are plant-based substitutes, defined here as products seeking full biomimicry of their animal-based counterparts (Good Food Institute 2019), and cellular agriculture, the production of animal products *in vitro* via tissue engineering and fermentation (Stephens et al. 2018). Over the last decade, proponents of these approaches have pinned planetary-scale promises to their technologies, evoking a vision of a “post-animal bioeconomy” (Datar 2015) that delivers a more sustainable, ethical, and healthy protein food system for all (Sexton, Garnett, and Lorimer 2019). This emerging industry has been described by Microsoft cofounder Bill Gates as a complete “reinvention of food” (Gates 2013). It has attracted multimillion-dollar investments from Gates himself and other high-profile figures in technology, venture capital, and entertainment. The year 2019 was particularly high profile for APs. Key moments included record sales in mainstream retail spaces (Forgrieve 2019), plant-based meat company Beyond Meat achieving one of the year’s best performing first-day initial public offerings (IPO) (Zhang 2019), and the first cultured meat product created in space (Smithers 2019).

This divorcing of animal products from animal bodies has created an unprecedented juncture in the ontological politics of food. Cowless milk and slaughter-free meat have not only created new questions and possibilities of what these foodstuffs *are* but also what they can and should be (Stephens 2013; Sexton 2018; Jönsson, Linné, and McCrow-Young 2019). Social scientists have begun to document the promises, technologies, regulations, and capital through which the meanings and materialities of APs are being constructed, and ultimately serving to materialize a future market (Stephens 2013; Jönsson 2016; Mouat, Prince, and Roche 2019; Sexton, Garnett, and Lorimer 2019). Mouat and Prince (2018) provide a valuable analysis of this market in formation. While the companies promote themselves as heavily mission driven, Mouat and Prince (2018, 316) highlight how markets continue to be centered in “the world that is being imagined and engineered” through these animal-free food ventures.

The specific role of *place* in the processes of AP market formation remains undertheorized.^1^ While recent AP activity is undeniably global in its movement of people, ideas, and capital, these extralocal networks have anchored to a small number of specific locales. At the institutional level, these include university laboratories and technology accelerators (Cohen 2013). At the (sub)national level, important hubs include Maastricht, Tel Aviv, Singapore, and—the primary focus of this article— Silicon Valley in California. Silicon Valley is especially conspicuous for its sustained concentration of AP activity. Many AP companies have physical headquarters in the San Francisco Bay Area, were founded through local tech accelerators, and have received a considerable portion of their funding from investors based in the region. The region has, at the time of writing, also hosted the largest number of industry-related conferences, and many of the sector’s supporting institutions (e.g., the Good Food Institute) have offices and personnel permanently based in the area.^2^

My chief contention in this article is that place, and specifically the place of Silicon Valley, has been a key and as-yet undertheorized mediator in the market formation of APs to date. I argue that it has been instrumental in two key ways: the first concerns the importance of being *physically* in place. The powerful place-myth of Silicon Valley as the “global innovation capital” (Piscione 2013, 46) has spurred a significant spatial migration of protein food activity to this industrial region, and the microspaces within it (e.g., start-up labs). This relocation has opened protein food economies to an entirely new wave of high-tech industrial actors with important consequences for the patterns and concentration of power in the global food system (Stephens, Sexton, and Driessen 2019). Yet, this is more than simple market capture played out across material space. My second analytical point concerns the importance of being *culturally* in place. I argue that the emergence of Silicon Valley as a leading hub of AP activity represents a fundamental pillar of AP ontological politics. Their development in *this* place has involved the reimagining of food in the information technology, venture capital-backed entrepreneurial image of Silicon Valley, at the same time APs have reimagined Silicon Valley as a leader in sustainable food futures. *Being there* (Gertler 2003) in spatial terms has crystalized a way of understanding food through the innovation models and practices of Big Tech and lent an important sense of *disruptive* credibility to this nascent sector. It has created a new geography of quality (Mansfield 2003, 2) for alternative foods based not on a sociospatial terroir of ingredients, as typical of other alternative food networks (AFNs) (Ilbery and Kneafsey 1998), but rather on what I term a *terroir of innovation* that has brought APs into being. As such, the promise and possibility of what APs can achieve has been significantly underpinned by where they and their economic geographies have emerged.

My analytical approach builds directly on keystone literatures in economic geography (e.g., Massey 1991a, 1991b; Storper 1997), and its agrifood subfield (Whatmore and Thorne 1997; Morgan, Marsden, and Murdoch 2008; Goodman 2011), that have defended the continued significance of place amidst the seemingly flattened geographies of a globalized world. Key to my argument is work that has viewed localized specificity through the lens of industrial culture, particularly that of Saxenian (1994) on Silicon Valley and Longhurst’s (2015) analysis of protective niches that inform my concept of innovation terroir. Taken together, I show how the industrial culture of Silicon Valley has shaped the politics of possibility (Gibson-Graham 2006; Guthman 2008) in the formation of the AP sector. This analysis is important for considering how AP development might be done otherwise (Broad 2019; Dutkiewicz 2019; Stephens, Sexton, and Driessen 2019) and what it means more broadly to look to Silicon Valley for solutions to global food security and other planetary challenges. I offer reflections on these points in the final section of the article.

## In Defense of Place: Embedding Food (Tech) Economies

My interest in the specificities of place in AP market formation follows an established direction of economic geography, both broadly and in the context of agrifood that has worked to rethink the processes and effects of globalization. Rather than think of place as now “transcended” (Coleman 1993) and altogether flattened into “one economic space” (Gibson-Graham 1996, 120), the *new* economic geography instead takes seriously the spatial unevenness of globalizing effects (Massey 1991b; Dicken, Peck, and Tickell 1997; Barnes 2001). This work blurs previous distinctions between economic and social life (Jackson 2002) and recasts globalization as multiple, unfinished, and open to alternatives (Gibson-Graham 1996).

The so-called “extraordinariness of food” (Goodman 2011, 243) provides rich examples of this hybridity and multiplicity despite the picture of placeless, market-driven global regimes put forward by political economic theories (Friedmann 1995). Assemblage thinking and the concept of embeddedness*—*which views economic activity as entangled in cultures, networks, and territories—are just two of the ways in which food scholars have enlivened geographic analyses of food economies and made sense of the prevailing significance and resistance of place in contemporary global foodways (Whatmore and Thorne 1997). From such viewpoints, agrifood capitalism does not run uninterrupted across surfaces, but rather snags in places, meeting resistance in the climatic contexts and biologies of the eaten (Goodman, Sorj, and Wilkinson 1987), and the visceral and cultural expectations of the eater (Guthman 2015; Sexton 2016, 2018). The continued significance of place is perhaps most salient in the resurgence of localism in AFNs. An attention to the localized places and ecologies of food production are frequently used to distinguish AFN products from their globalized, industrialized counterparts (Ilbery and Kneafsey 1998; Goodman, DuPuis, and Goodman 2012). By grounding food in place, such attentiveness imbues a sense of care from both producer and consumer for the otherwise hidden networks of the food system. It can create what Mansfield (2003, 3) terms “geographies of quality” that facilitate regional comparative advantage in the face of industrialized food production.

The AP sector in Silicon Valley offers a distinct case of the importance of place in alternative food economies. On the one hand, this is a familiar story of an AFN using place in its market formation to set AP ventures *there* apart from competitors elsewhere. Yet unlike AFNs, where place is made significant through an embedded geography of quality based on social (e.g., traditional skills) and physical attributes (e.g., soils, flora, climate), the foodstuffs I explore are instead made distinct entirely through an embedded *culture* of high-tech innovation. More than, or indeed in spite of, California’s global reputation for producing quality, alternative foodstuffs (Guthman 2014), the importance of APs being in Silicon Valley draws upon a different kind of localized assemblage, one based in technoindustrial networks and a shared spatial imaginary that promotes this place over all others as the global capital of innovation (Piscione 2013). The geographies of quality that have mattered for APs to date, then, are less attentive to the terroir of ingredients and more on the *terroir of innovation* that brings them into being. A key consequence of this reimagining of food as tech has produced new and influential spatial realities of protein food production that have led to Silicon Valley, a technoindustrial region previously disinterested in food ventures, becoming a leading hub in the race to fix food in the Anthropocene (Sexton 2018).

This spatial politics is about more than just a reorganization of food economies across territory. As the reviewed literatures demonstrate, to be in place is to be embedded within its cultures, networks, and politics. To *do* food in Silicon Valley thus comes with specific inflections of its locality as a leading center for high-tech industry, which in turn bring significant consequences for the pathways of possibility open to AP development.

## Innovation in Place: Silicon Valley as a Culture of Doing

To think of innovation as possessing a terroir or embeddedness draws in part on geographic studies of innovation that have sought to make sense of spatialized competitive advantage within industries. From the centuries-old leather-making regions of Italy (Randelli and Lombardi 2014), to tomato growers in Toronto (Blay-Palmer and Donald 2006), and indeed the high-tech hub of Silicon Valley (Porter 1998), the spatial underpinnings of innovation practices have been well documented by economic geographers. Yet despite their focus on localized scales, the specificity of place often remains undertheorized in these debates. It is often treated as a passive backdrop to proximal institutions rather than considered an active mediator in the doing of industrial work. Through such readings, innovation is presented as a predictable product of “input-output relations and material linkages” (MacKinnon, Cumbers, and Chapman 2002, 296) and replicable across geographies, as demonstrated in the multiple attempts to create additional *Silicon Valleys* in other sites around the world (Saxenian 2005).

To make better sense of how Silicon Valley is shaping the practices of AP innovation requires more hybrid understandings of place and innovation. The work of AnnaLee Saxenian provides a helpful starting point. Her ground-breaking book comparing the different trajectories of Silicon Valley and Route 128—the latter being a similar yet less economically prosperous high-tech industrial region in Massachusetts—makes a compelling case for the role of local determinants in shaping the activities of these industrial regions in the face of parallel political and economic pressures (Saxenian 1994). Her focus on the interconnectedness between local culture, industrial structure, and corporate organization departs sharply from the discrete models of previous agglomeration theories: it does away with the notion that the correct assemblage of generalized ingredients in any given locale will inevitably result in successful regional growth. Instead her work takes seriously the *character* of place (Massey 1991a) and its key role in shaping how industrial practices are designed and conducted. It is these local differences, she argues, that can influence the capacities for innovation and lead to regional comparative advantage.

Unlike Saxenian’s (1994) core focus, my interest in this article is not to measure the regional advantage or success of Silicon Valley’s recent turn to APs. Rather I look to her work for its emphasis on an identifiable local culture of doing innovation and the relationship between this culture and the region’s industrial practices. In her analysis, Saxenian highlights the so-called softer factors often disregarded as superficial in the models of previous economic geography (cf. Schoenberger 1997). For example, she notes how the differences “between ‘laid back’ California and the more ‘buttoned up’ East Coast” have manifested in the regions’ respective industrial organization and ability for adaptation (Saxenian 1994, 2). Others have made links between Silicon Valley’s renowned frontier imaginary—tracing this back to the Californian Gold Rush (Matthews 2003) and the Bay Area radio industry in the late nineteenth century (Sturgeon 2000)—and its propensity for pioneering leaps over incremental innovation (Storper 1997). The counterculture movement of the 1960s and ’70s is also a strong influence on the region’s spatial identity, especially as it relates to food. During this time, the Bay Area was brimming with student protest, psychedelic experimentation and back-to-the-land communes designed to “recover the ‘true self’” from “technocracy and the technocratic control of human bodies and minds” that the Cold War era had come to represent (Rycroft 2007, 619; see also Turner 2010). Collectively, these histories have come to manifest in what Weiner (2016, 297) describes as a culture of “brutal optimism”:

“Brutally optimistic” is how one local put it. Anywhere else in the country, he explains, your new idea is met with an avalanche of reasons why it won’t work; in Silicon Valley, it’s met with a challenge. *Why don’t you do it? What are you waiting for?* Brutal. (author’s emphasis)

It is these intangible dimensions of Silicon Valley’s industrial culture (cf. Schoenberger 1997) that I examine in relation to its local AP sector.

### Possibility in Practice and Place

Exactly how broader place-myths come to infuse regional innovation practices is less clear in the literatures reviewed above. Here we can turn to Longhurst’s (2015) recent study of Totnes in the UK to help reveal these linkages. Longhurst shows how space is an integral component in the ability of Totnes to act as a protective niche for a particular variety of innovation—what he refers to as *alternative innovation*.^3^ As well as the importance of physical space (i.e., geographic proximity), Longhurst points to the role of *sociocognitive space* (how a place is perceived) in fostering experimentation in Totnes. He outlines how this particular type of space is created in three ways: first, by producing “ontological and epistemological multiplicity”; second, by producing “spatial imaginaries”; and third, by providing “ontological security” (Longhurst 2015, 189).

The first dimension reflects a distinct “culture of ‘credulousness’” within the Totnes milieu that produces “the socio-cognitive space for experiments to emerge by stretching the socially accepted (and constructed) boundaries of possibility” (Longhurst 2015, 190). This stretching of possibility, Longhurst argues, is a consequence of Totnes’ history as a place of diverse, alternative epistemologies and ontologies. Like the Bay Area, Totnes is a hotspot for New Age beliefs and radical politics. The historic– geographic embeddedness of Totnes is thus conceptualized by its inhabitants as providing a sociocognitive space where multiple radical ideas can coexist and flourish unhindered by normal perceptions of possibility.

The second dimension relates to how the physical landscape of Totnes plays a role in the area being viewed as a *good place* for innovation. Inhabitants described the utopian landscapes of Totnes, both in their aesthetic natural beauty and the many visible successes of experimentation throughout the area. For these reasons, Longhurst found a similar value attached to being *there* (Gertler 2003)—that is, physically present within the milieu—that is often expressed by Silicon Valley actors.

Underpinning these dimensions, the third describes the like-mindedness of people in the Totnes milieu who share a common goal of seeing and creating the world differently. Key here is the practical and moral support for innovative ideas among the area’s inhabitants, where people help each other to think, create, and even live in ways outside of dominant social and cognitive norms (e.g., illegally living on their land). Longhurst identifies the normalization of these nonmainstream practices within the locale as a fundamental component in shaping the alternative variety of innovation enacted in the area.

### An Alternative Milieu?

Attention to the sociocognitive spaces that concentrate within localities reveals the power of place to cultivate particular ways of doing innovation as well as to instill a sense of normativity around such practices (i.e., what qualifies as innovation). Exploring these dynamics builds directly on existing accounts of Silicon Valley’s industrial culture by taking seriously the power of beliefs about a place to shape the politics and possibilities of practices within its locale (cf. Gieryn 2018). This work is useful, therefore, to not only understand how and why APs have come to be physically in place within Silicon Valley but also how the powerful place-myth of the region has resulted in a distinct cultural emplacement of their development.

Yet more specifically, I use Longhurst’s work to attend to the uncritical celebration of Silicon Valley’s so-called alternative or disruptive innovation that prevails in academic and popular commentaries. Such accounts often lack critical engagement with the political underpinnings that shape what is possible within Silicon Valley’s innovation culture (Morozov 2013). As I will show, being both physically and culturally *in place* as a Silicon Valley entrepreneur not only shapes the likelihood of business success but also the pathways open to a venture’s technological and economic development. To be in place in these ways is to ascribe to a specific approach of doing innovation within the Silicon Valley locale—what Goldstein (2018, 10) terms “non-disruptive disruption”—that remains loyal to capital-friendly practices and high-tech products. Spigel (2017) contends that entrepreneurs “by definition must transgress existing norms and structures in order to succeed,” yet I found that such transgressions within the Valley-based AP sector were actively discouraged by the regional culture; indeed, metrics of success were rather predicated on their absence. For an AP entrepreneur to eschew the region’s cultural norms risks disconnection from its coveted networks of social and financial capital, and disqualification as a valued contributor to the local disruptive landscape.

In contrast, then, to Longhurst’s (2015) observation of ontological and epistemo-logical *multiplicity* in Totnes, I witnessed a distinct *singularity* in the innovation pathways open to AP ventures in Silicon Valley. The following subsections use Longhurst’s categories with this key amendment to explore the dominant culture of high-tech innovation in Silicon Valley and how it has come to imprint on its AP sector.

### A Note on Methods

The data analyzed in this article was collected during four separate research visits to the Bay Area between 2015 and 2019. Forty-one semistructured interviews were conducted with key AP stakeholders directly working in the commercial development of cellular agriculture and plant-based products and those supporting the sector through financial investment, scientific research, and advocacy work. All of the companies in Table 1 were approached as representative (though not exhaustive) of the leading cellular agriculture ventures working in the Bay Area during the period of research. My participants included founders, senior employees, and researchers from these and other industry-leading AP ventures, including plant-based companies, in the Bay Area, Southern California, and New York City.

Additional interviews were also conducted with fund managers of venture capital firms who have made leading investments in the Bay Area AP sector, ranging from corporate ventures of multinational food companies to private, mission-driven firms; a leading US nonprofit organization supporting the AP sector through public engagement, research fund-raising, and industrial lobbying; and members of two citizen science laboratories working on the Real Vegan Cheese open-source project. The interviews ranged between twenty minutes to half a day with the majority lasting between one to one and a half hours. These discussions included a number of follow-up meetings with participants during my subsequent fieldwork visits to the Bay Area between 2015 and 2019. My analysis also draws on observations of key industry and research conferences in the Bay Area, Boston, Washington, DC, and Northern Europe over this time period. This combination of qualitative data was collated and analyzed for key trends, themes, and insights from those directly involved in the early years of AP activity within Silicon Valley. The article thus contributes original empirical and conceptual insights on the sector during its formative years in a geographic place—Silicon Valley—that has until recently been a less studied chapter of the AP industry.

## Spatial Imaginaries: The Importance of Being There

Stephens, Sexton, and Driessen (2019) identify a second wave of cellular agriculture development in the early 2010s that signaled a new direction of institutional support away from academic institutions and toward high-tech, private industry. This institutional shift was distinctly geographic and saw the beginnings of AP concentration in Silicon Valley. The first cell-cultured burger, produced in the Netherlands, became one of the earliest connections between cellular agriculture and Silicon Valley through the project’s primary investor, Google cofounder Sergey Brin. In 2013, the same year the burger was publicly unveiled, Modern Meadow became the first cultured meat start-up to enroll in a tech accelerator, choosing the Singularity University program in Silicon Valley with its mission to support “breakthrough technologies to address humanity’s global challenges” (Stephens, Sexton, and Driessen 2019, 6).^5^ As of August 2019, there were forty-three publicly announced cellular agriculture companies and one citizen science project (Real Vegan Cheese) in five continents producing a variety of edible protein products.^6^ Of these forty-four ventures, thirteen are based in the San Francisco Bay Area, California, constituting the largest concentration in one geographic place to date (see Table 1).

The cellular agriculture scene of Silicon Valley has emerged alongside a burgeoning sector of new plant-based analogue start-ups. The US West Coast was already home to household plant-based brands (e.g., Tofurkey in Oregon, Miyoko’s Kitchen in northern California), yet the founding of Beyond Meat (Los Angeles), JUST, and Impossible Foods (Bay Area) between 2009 and 2011 saw a distinct departure in technological approach, branding, and business model. JUST and Impossible Foods are still headquartered in the Bay Area, and all three companies received early investment from venture capitalists (VCs) and high net-worth individuals connected to Silicon Valley. These companies are frequently held up as the original movers among their industry peers in the latest generation of plant-based biomimicry on account of their pioneering multimillion dollar investment rounds, record-breaking market debuts, and transformation of plant-based substitutes into products now increasingly purchased by nonvegans (Settembre 2019).

Most of the AP founders I interviewed told me they had moved to Silicon Valley specifically to start their companies. When asked why, all made reference to the region’s concentrated niche of institutions and expertise that made it considerably easier to begin a start-up compared with other parts of the US. I asked one local plant-based company whether they thought the venture could have grown as quickly if it had started in another place:

There might be a few places we could have started in like Austin, maybe Boston possibly, but the beautiful thing about San Francisco is we have such access to VCs—they’re everywhere and they come here and feel our energy and see what we’re doing. It’s been big with the press; it’s also been big with recruiting so there’s three levels—that’s why San Francisco is such a hotbed of innovation because those three things are everywhere.^7^

Access to venture capital with high-risk tolerances was a recurring theme in my conversations with AP company founders. The relatively small academic and government grants that had supported some of the first wave AP ventures ultimately proved inefficient to take on the scale of risk and investment needed to bring early proofs of concept anywhere close to market readiness (Stephens, Sexton, and Driessen 2019). To do this, capital with much higher risk tolerances and a hunger for the next moonshot idea was needed, prompting a geographic movement of AP founders to Silicon Valley.^8^

The quote above highlights two other regional resources that were emphasized by many interviewees: an established knowledge economy and connections with mainstream media. This constellation of three levels—capital, talent, and media—echoes the neoclassical analyses of the region with an emphasis on spatial proximity (e.g., Porter 1998). Greater availability of commercial laboratory space was also cited by several participants. An investor recounted an experience by one of their portfolio companies finding it much easier to rent out lab space in the Bay Area—“they could move in tomorrow”^9^—compared with their home city of Chicago, and the cofounder of another AP company attributed their move West to the greater availability of private lab space through local tech accelerator IndieBio.

Without dismissing the advantages of the region’s specific concentration of resources, this does not capture the whole picture. In my interviewees’ descriptions of the region’s institutional ingredients, it became clear that the importance of being there was for more than simple logistical ease. Some referred to quasi-spiritual benefits of being in the region, where investors can “come and feel our energy,” and founders can arrive with just an idea and build on the Schumpeterian entrepreneur-spirit (Schumpeter 1934) of the milieu’s previous industrial successes. This latter point was conveyed by Clara Foods cofounder Arturo Elizondo in a 2015 media interview. Describing his move to the Bay Area, he stated, “I was so into food tech, and I knew DC wasn’t the place for that, so I figured I should probably be in San Francisco to pursue my passion. A week later, I booked a one-way ticket, no job, no place to stay” (New Harvest 2015). The vision of following in the region’s high-tech successes was directly referenced by another AP company interviewee who stated, “as Uber is a leader in transportation, as Airbnb is leading disruption in hotels, we want to create that with food.”^10^

I was told by an investor that mainstream media also typically attribute more prestige to ventures based in Silicon Valley compared with other parts of the world:

What Silicon Valley are very accustomed to is getting their companies noticed. They can pick up the phone and call someone from the New York Times and the next day there’ll be a story appearing in there about their company … If a VC in, let’s say, Ecuador decided that they wanted the same attention drawn to their investment in Ecuador it wouldn’t make it on the front page of the New York Times unless it’s truly remarkable, but in Silicon Valley it’s a little bit easier to push that to the front page because there’s some notoriety there, there’s some cachet.^11^

Another investor stated that the cachet afforded Silicon Valley–based companies has led VCs in the region to rarely look beyond their locality for the next big venture; they are “typically just investing in [their] own backyard.”^12^ Moreover, the ability to access local institutions, such as laboratories and tech accelerator programs, was attributed both to their greater number compared with other parts of the US and their openness to atypical ideas: “It’s a forward thinking, high-tech, early adopter, food conscious-type of place … it was a no brainer to move here.”^13^

Being enrolled in the region’s prestigious accelerator programs has also worked to make AP start-ups more visible within the Silicon Valley scene and promote them as credible ventures for others to get excited about and, ultimately, invest in. These benefits were described by a cellular agriculture cofounder enrolled at IndieBio during 2015: “It’s the number one biotech accelerator in the world so it definitely lends—you know, whenever someone can validate that this is an investment that somebody else they believe in [i.e., IndieBio] has made, that adds credibility, just instantly.”^14^

Another alumnus described the “king-maker” power of local accelerators such as IndieBio and Y-Combinator; he told me they are “very well respected amongst Silicon Valley investors,” and their Demo Days are some of the most exclusive tickets in the region, if not the world, for discovering the next Facebook or Uber.^15^

It became apparent that a distinct and powerful spatial imaginary is at work across these institutional levels that positions Silicon Valley as *the* place where radical innovation occurs and is encouraged. This view extends beyond the practical components that facilitate the business of doing innovation to also include the area’s perceived cultural openness to alternative ideas—what Longhurst (2015) refers to as ontological and epistemological multiplicity—and being able to work within the ontological security of the region’s industrial culture. Many interviewees referred to the long-held enthusiasm for countercultural experimentation and entrepreneurialism in the San Francisco Bay Area and more broadly across California (Kirk 2007; Turner 2010). Much like Longhurst (2015) observes in Totnes, these countercultural histories combined with the pleasant weather, natural beauty, perceived open-mindedness, and local examples of successful high-tech companies (e.g., Apple, Google) have all served to fuel a spatial imaginary of the region as a place where innovation happens and succeeds, both in economic terms and in radically shifting paradigms of practice. These aspects have collectively formed a distinct terroir of innovation that sets AP ventures there apart from those elsewhere. Confidence in what APs can deliver for people, planet, and financial profits has, as such, been hugely shaped by *where* they have emerged, making place a pivotal factor in their ongoing ontological politics.

## Being Culturally in Place: Not *All* Ideas Welcome

*You have to be innovative in the right ways a lot of the time*. (Cofounder, cellular agriculture company)^16^

### Learning the Norms

During my time engaging with the AP sector in Silicon Valley, I observed quite a different picture from the ontological and epistemological multiplicity Longhurst (2015) describes in Totnes and that is commonly attributed to Silicon Valley. Through my stakeholder interviews and observations of institutional practices, it became apparent that the business of disruption was rooted in a highly defined structure of rules and established pathways. As one AP company founder told me, there are “right ways” to be innovative in the region and to succeed required “learning the norms”—a process that remains, he noted, embedded in distinct heteronormative, gendered, and racial biases, and had been considerably more accessible to him as a cisgendered white male (see Shih 2006).^17^

A key norm exhibited by the region’s AP sector is the distinct homogeneity in its organizational models. For example, all but one of the AP ventures based in the region have so far evolved as private companies. The majority were founded as such, while others began in academic contexts and have since evolved into commercial enterprises. As discussed above, lack of public funding has played a key part in shaping these trajectories. Yet, many AP founders also attributed this trend to the common ideological perception in the region’s high-tech community (which many of them also personally shared) that the private sector is the best mechanism for innovation. As one cofounder of a cellular agriculture company stated, “I’m sorry but it’s over—academia is not doing it [innovation], government’s not doing it. It’s companies.”^18^ A similar sentiment was shared by Joshua Tetrick, CEO of plant-based and cellular agriculture company JUST, in a 2016 interview with *WIRED* magazine: “The more I drilled into food, the more I thought capitalism could be used to reorient the system” (Solon 2016).

Tech accelerator programs have played a key role in the AP sector’s steer toward private companies. IndieBio programs are explicitly designed for applicants to go from “bench to market” within four months.^19^ During the opening speeches at a 2016 Demo Day, IndieBio Program Director Ryan Bethencourt explained how this mission starts from day one of the program when each team is asked “how long until you have a product that makes money?” The necessity to adopt for-profit models to gain interest from the region’s industrial community was further demonstrated by an AP company founder when discussing a nonprofit venture they had recently met:

[H]e’s inventing this cocoon that allows you to plant trees in the desert and they’ll grow happily and productively. But the problem with that is that even though it’s a cool idea and he’s already done pilot testing and all of that, no VC is going to fund his company. They’re not going to get the returns they need; I mean this is more like a non-profit, maybe they’ll make a few million but no VC is going to invest in them.^20^

We see here a limit to the ideas that are taken seriously and given support by the region’s high-tech milieu: where “cool ideas” with tested proofs of concept, yet lacking the promise of financial returns, are overlooked by the dominant innovation culture of the region. Many of the investors I spoke with had mission-driven investment theses— that is, they had interests in *doing good* through their portfolio companies—but ultimately were based on models of conscious capitalism (Mackey and Sisodia 2013) that required attractive financial returns and exit strategies. In my conversations with investors I was frequently told that their investments in AP ventures were “not a donation.”

The Real Vegan Cheese project (RVC) provides a striking comparison of the different trajectories that for-profit versus nonprofit AP ventures have experienced in the Bay Area to date. Like private companies, such as Perfect Day Foods, RVC is seeking to remove animals from milk production by genetically altering yeast cells to produce casein, a milk protein.^21^ However, RVC’s model is entirely based on open-source, community-led science supported through crowdfunding campaigns and part-time volunteers. The project was started in 2014 following an IGEM (International Genetically Engineered Machine) competition and is based across two community science labs in the Bay Area: Counter Culture Labs in Oakland and BioCurious in Santa Clara. The project’s mission echoes the ethos of these community labs through its work to develop the basic science of cultured dairy production and make their protocols available via a public Wiki page. Two of the regular volunteers explained this approach was not to preclude companies being founded from the work, but rather to limit the concentration of ownership within the sector:

RVC1: “So if someone wants to start a company from this work that’s great, but it’s just that they can’t be the only one.”

RVC2: “The idea is to make sure that no one can take this and claim it for themselves, because that’s the basis of the group. [We] split from a lot of the companies that are in this area because in order to get investment capital you need to have a patent as the investors don’t feel comfortable investing in a company unless they have some competitive advantage in the space that they’re trying to enter.”^22^

They described how many of the AP companies in the region have developed aggressive patent strategies typical to the region’s high-tech industry and biotechnology more generally (Parry 2004). Some companies attempt to own their entire production process from ingredient to final product (referred to as the *full-stack model*). While sympathetic to the overall outcome of removing animals from food production, it was clear the RVC volunteers were concerned about the pathways being taken by many in the sector and the political–economic implications this posed for democratic science and knowledge: “[patents] will block other people from using certain processes, but that’s just the nature of how the system works here in the Bay Area”^23^

Knowledge of RVC was partial among the investors I spoke with; some had heard loosely about the project, and only one had visited the labs in person. Of this selection, most appreciated that the project had worthy and shared goals but that their approach was a “difference of opinion”:

I give them a lot of credit because their interests are very much aligned with our investment thesis, which is moving the category forward. It’s just what is the right means to do so. RVC is a different approach … I think I like the pathway that we’ve chosen through investments. But it’s [RVC] another pathway.^24^

RVC represents a different pathway of AP innovation that is valued by some in the sector insofar as it is helping advance technical knowledge, but it remains unbackable by the region’s high-tech community due to its choice to operate beyond the Valley’s cultural norms. RVC’s refusal to exist as a for-profit venture based on proprietary knowledge has precluded it (by choice) from access to venture capital, and it is rarely mentioned in key AP reports, industry events, or media coverage as being part of the region’s disruptive landscape. In this way we can conceive of RVC as physically in place but culturally out of place in the Valley milieu. While a multiplicity of innovation pathways was acknowledged as possible by some in the AP sector, it was clear that the route to success was viewed as decidedly more singular, reflecting the region’s specific ontological and epistemological *singularity* of what qualifies as innovation and how it is practiced.

### Food-as-Software

The weight afforded to patents in the current AP sector is in part an artifact of the hightech culture of innovation that evolved during the semiconductor boom in Silicon Valley in the 1970s and ’80s. During this time, a culture of aggressive patenting developed, creating an environment of high-stakes “patent portfolio races” (Hall and Ziedonis 2001, 104) as rapid technological development forced firms to protect as many pieces of the innovation-scape as possible, particularly as most computing products relied on technologies from across the semiconductor industry. Yet the emphasis placed on intellectual property for AP ventures also reveals the necessity of aligning the world of food with the *language* of Silicon Valley. While much is made in popular imaginaries of the region’s thirst for new industrial frontiers, my interviewees described a far more cautious and conservative culture among the region’s investment community. Part of their thesis of investing in “their own backyard,” as one interviewee described, has also been to invest in familiar types of ventures and industry segments. For example, many VCs have clearly defined sectors and stages of companies they are interested in funding. Information technology (IT) largely remains the common theme across these categories—that is, often the novelty that Silicon Valley investors seek is the application of familiar technology platforms to new markets such as Airbnb with the hospitality industry.

A challenge faced by early AP ventures was that food was neither an attractive nor familiar market for Silicon Valley investors. As one investor told me, it was perceived as “a boring and unsexy industry” and “too real” by the region’s investment community:

The real challenge is that venture capital tends to stay in the world of software, where you want to make “2” you hit a button, you want to make “3” you hit a button. Whereas things that need to interact with the physical world, you need to physically make something. Venture capital has tended to stay away from that. They [Silicon Valley VCs] did get really burned in the clean tech boom and so there’s also some hangover from that, where people are like yeah, we tried to venture out into the real world, maybe we should stick to the virtual world.^25^

While Silicon Valley has an extensive ecosystem of software-based ventures related to food (e.g., recipe apps, delivery services), the AP sector presents a much messier crossover of technology and food: their products are not based in software and data alone, but rather encompass the materialities of food itself, along with its associated regulations, politics, cultures, and tight profit margins. Attempts to simply transfer the IT-based VC model of doing innovation to APs has imposed a set of norms that have proved challenging, such as the need to establish defensible assets that can be protected under patent law, the need to quickly prove the scalability of production, and the intense time pressures to create material products. Many AP founders told me these expectations are relatively easier and quicker to achieve when working in the IT-based realms of algorithms and products not intended for bodily consumption.

According to a Silicon Valley–based VC speaking at an industry event in San Francisco in 2015, the defensible asset for food companies and products has historically been the brand. Yet despite a brand being difficult to replicate, he highlighted that at the start-up stage “there isn’t much of a brand yet, so how do you put a huge valuation on that?”^26^ In recognition of this, many AP ventures have made conscious adaptations to their innovation trajectories, both materially and discursively, to ensure they meet investor requirements of scalability and patentability. Two separate interviewees offered insights on these decisions:

When we’re talking to an investor the focus shifts to technology, to what we’re doing in a lab here. Because the investor sees a “food company”—that’s not scalable, that’s not huge, it’s not going to be a unicorn.^27^ You know, “is it worth my time?” But when you talk about building databases, computational biology like predictive models of screening plants, then they think wait, that’s something that’s scalable—like 400,000 plants in the world, you guys could license this out. That is what gets them excited more than just [food] as a product.^28^

These companies think of themselves as food technology companies. They call themselves a *platform*: “we’re building a technology platform that’s agnostic about cell line culture.” And it’s like woah, no you’re not, you’re a farm, like what?! Even thinking of themselves as technologies I think has all of these consequences … the ethos of what you’re producing and why and how you’re producing it, and for whom, is very radically different … the design principles, investment theses, business plans and models: when you start thinking of yourself as a technology company therein lies the potential challenge. And I’m not sure what to do about it, because in many regards you need to do that for investors and the investors you’re going after.^29^

These adaptations have been fundamental to bringing the world of food into the institutional language and models of Silicon Valley–style innovation and have been a distinct feature in the subsequent marketing of the region’s AP companies: for example, when Beyond Meat launched a second iteration of their plant-based burger in 2019, it was promoted as the *Beyond Burger 2.0*, a clear nod to the terminology commonly used by Apple and other IT companies to launch new generation versions of their products. This framing took the potentially unsexy process of recipe development common to new food companies and repackaged it in the more exciting language of software development.

Media coverage presented this approach as signaling a “new era” of meat production where “new versions of the product are constantly being tweaked, updated, and released. In short, we are entering an era where food is becoming more like software” (Albrecht 2019). This analogy formed the basis of an influential industry report by RethinkX published in 2019 in which it coined the term *Food-as-Software* to describe the new era of post animal food products. In their words, food-as-software is “the new model of food production and consumption that adopts certain principles of modern computing” (RethinkX 2019, 10). The proposed benefits of this reimagining of animal-food production echo those in the narratives of AP companies, namely, exponentially increased efficiency and superiority over the *broken* and *stagnant* models of conventional livestock systems (Sexton, Garnett, and Lorimer 2019):

Continual iteration means modern food products will improve rapidly, both in functional attributes and in cost—just as version 1.0 hits the market, companies will be working on version 2.0 already, then 3.0, and so on, with every version superior and cheaper than the last. This rapid improvement is in stark contrast to the industrial livestock production model, which has all but reached its limits in terms of scale, reach, and efficiency. (RethinkX 2019, 17)

To make food attractive to the institutional landscape of Silicon Valley, it was recognized early on by AP founders that their products had to be framed as technology first and food second. They had to become culturally in place by fitting seamlessly within existing VC portfolios that favor high-tech ventures, as well as promise to deliver the same innovation and funding cycles expected of software start-ups. Reimagining food as technology has thus worked to align AP ventures, in the way the region’s high-tech community conceives of and conducts innovation; it has tamed the material messiness of food and made it palatable to the ontological and epistemological *singularity* of the region, which privileges for-profit, high-tech models as the most effective and ultimately lucrative model of innovating.

## Reinventing Food Silicon Valley–Style

The aim of this article has been to emplace AP activity by taking seriously the role of place in shaping the ontological and political–economic trajectories of this emerging alternative food sector (cf. Gieryn 2000). Silicon Valley has become a leading geographic hub of AP activity at a formative point in this nascent sector’s evolution. This spatial concentration has in part been driven by logistical reasons (e.g., access to capital). Yet, as the article has shown, the importance for AP founders to be physically in place within the environs of Silicon Valley has been motivated by broader spatial imaginaries of the region as *the* place of disruptive innovation. Particularly in the early days of the AP sector, being based within Silicon Valley’s specific terroir of innovation was seen to provide a unique protective niche of credibility and credulity for these nascent ventures to be noticed and jump-started as businesses. Their ontological realities have, as such, been underpinned by a distinct spatial politics: *where* they are materializing has shaped ideas of *what* they are and might achieve.

For APs to be physically in place has also involved an encountering with a specific culture of logics and practices of the technoindustrial region of Silicon Valley. To succeed at doing protein food in this locale has required it to become *technology*—it has required a choice by ventures to become culturally in place and reimagine food through Silicon Valley’s image of for-profit, high-tech innovation. As the case of RVC demonstrated, alternative innovation pathways are present within the region yet are typically viewed as outside industrial cultural norms; as such, they have remained disconnected from the mainstream channels of social and financial capital in the Valley and an overlooked component of the region’s so-called disruptive landscape. Such insights reveal the political situatedness of innovation practices (Morozov 2013) that has consequences not only for the ontological politics of APs but also more broadly to the question of looking to Silicon Valley for solutions to global food problems. To do so is to venture into the heart of the culture of “non-disruptive disruption” (Goldstein 2018, 10), where markets remain resolutely undisrupted, and planetary crises, such as food insecurity, become another frontier of for-profit technofix solutions.

Moreover, reinventing food Silicon Valley–style in these ways has reimagined who is best to push this so-called new era of food forward (Sexton, Garnett, and Lorimer 2019). It has opened the political economies of protein food to tech accelerators, entrepreneurs, and VCs, creating new opportunities for high-tech industries to expand into new markets. Protein food promises a new avenue for high-tech appropriation of the biological, with some forecasting the ultimate outflanking of nature as food is rendered entirely software. In a present where Silicon Valley companies increasingly mediate our social lives, domestic spaces, and personal data, it is perhaps not surprising that Big Tech is now inviting itself to our dinner tables. Such trends raise urgent questions concerning how food systems will come to be organized over the coming years, the livelihoods and landscapes they support, and the ethical consequences of high-tech ownership extending yet further into everyday life.

Taking this work forward, the contemporary and distinctly emplaced moment in which the worlds of (protein) food and Big Tech are meeting offers economic geographers many timely avenues for continued critical analysis. The specific confluence of food, technology, and place that APs represent speaks directly to core debates within economic geography, yet also invites the discipline to extend into new cross-disciplinary conceptual ground. Engagement with science and technology studies offers much for further elucidating the particular conditions of Silicon Valley’s innovation terroir (cf. Barnes 2008; Truffer 2008; Furlong 2010). In the vein of Latour and Woolgar’s (1986) laboratory ethnographies, there is great scope for similarly emplaced research within the microspaces of Silicon Valley’s AP community (e.g., tech accelerators and start-up labs) to gain further insight on how the regional culture is translated through its institutions and, in turn, becomes imprinted on its everyday innovation practices.

Economic geographers are similarly well placed to further unpack the place-based conditions that enable certain economic models to thrive over others in innovation-scapes like Silicon Valley and the implications this has specifically for creating alternative food systems (Watts, Ilbery, and Maye 2005). This work would complement recent commentaries that have challenged the Silicon Valley–style model of innovation both in relation to food and other contemporary issues (e.g., Morozov 2013; Srnicek 2017; Daub 2020). Alternative visions of an AP industry based on a framework of “food tech justice” (Broad 2019) and socialized models of finance and public ownership (Dutkiewicz 2019) have recently been put forward, and the industry itself appears to be grappling with the pitfalls of its emplacement within Silicon Valley:

What’s common in Silicon Valley is that you move fast and break things, but that is an awful way to approach making food. Consumers have an expectation that food is safe and so from a regulatory point of view we want to work with all stakeholders, all producers, everyone we can. (CEO of Memphis Meats, quoted in Watson 2018)

Such comments speak to a distinct resistance of food’s natures and incumbent networks to the complete reinvention of food Silicon Valley–style. The specifics and processes of this resistance offer further themes for future geographic research. A fruitful avenue would be to engage economic geography with materialist-focused work in food geography (e.g., Roe 2006; Goodman 2016; Sexton 2018) and critical agrifood scholarship (Goodman 2004; Guthman 2015) that has documented similar examples of the material and reputational limits that prevent, or at least complicate, the capitalist appropriation of food. Bringing these debates together promises new insights on the particularities that set food apart from other commodities (Goodman 2011) and that, in the case of APs, have remained a key barrier to their complete rendering as software. Moreover, there are key political–economic implications of food’s resistance to Silicon Valley reinvention that warrant further critical study, particularly concerning what exactly is being disrupted and left intact by recent AP activity (Sexton 2016, 2018), and who will continue to control (protein) food in this so-called new era. There is much scope for economic geographers to continue examining these and other unfolding consequences of Silicon Valley’s recent turn to food, and the politics of possibility this opens and closes for realizing sustainable, equitable food futures.

## Figures and Tables

**Table 1:** Publicly Announced Cellular Agriculture Ventures Based in San Francisco Bay Area, California as of August 2019

	Company	Product
1	New Age Meats	Pork
2	Finless Foods	Seafood
3	Blue Nalu	Seafood
4	Wild Type	Seafood
5	Clara Foods	Eggs
6	Geltor	Other^4^
7	Wild Earth	Pet food
8	Memphis Meats	Multi—meat
9	Missions Barns	Multi—meat
10	JUST	Multi—egg, meat, seafood
11	Perfect Day	Dairy
12	New Culture	Dairy
13	Real Vegan Cheese	Dairy

Source: The New Protein Industry V. 2.7: Olivia Fo Caane, www.newprotein.org.

## Footnotes

1 An exception is Jönsson, Linné, and McCrow-Young’s (2019) examination of national laws in shaping where cultured meat products may eventually come to market.

2 Conferences have included the Good Food Conference (2018, 2019), New Harvest (2016), Cultured Meat Symposium (2018, 2019).

3 Defined by Longhurst (2015) as innovation beyond mainstream political, economic, and sociocultural models.

4 Geltor, formerly Gelzen Inc., describes itself as a “conscious biodesign company creating the world’s most advanced designer proteins,” such as animal-free collagens, for food, beverage and cosmetics industries (www.geltor.com/about-us/).

5 Modern Meadow has since focused on cultured leather production and is based in Brooklyn, New York.

6 AP company stats as reported in *The New Protein Industry V. 2.7: Olivia Fox Cabane* (www.newprotein.org).

7 Fieldwork interview 2015.

8 A small number of university-based research has continued throughout this *second wave*, and since 2018 there have been high-profile public investments made by national governments, including Singapore, Israel, and Japan (Stephens, Sexton, and Driessen 2019).

9 Fieldwork interview 2019.

10 Fieldwork interview 2015.

11 Fieldwork interview 2019.

12 Fieldwork interview 2019.

13 AP company, fieldwork interview 2015.

14 Fieldwork interview 2015.

15 Fieldwork interview 2019.

16 Fieldwork interview 2019.

17 Fieldwork interview 2019.

18 Fieldwork interview 2015.

19 Fieldwork event, IndieBio Demo Day 2016.

20 Fieldwork interview 2015.

21 https://realvegancheese.org/.

22 Fieldwork interview 2019.

23 Fieldwork interview 2019.

24 Investor, fieldwork interview 2019.

25 Fieldwork interview 2019.

26 Fieldwork event, Bon Appétech Conference, San Francisco 2015.

27 A *unicorn* in Silicon Valley parlance refers to a company that has reached a valuation exceeding US $1 billion. Only a small percentage of companies go on to reach this coveted target; examples include Google, Facebook and Airbnb.

28 AP company, fieldwork interview 2015.

29 AP commentator, fieldwork interview 2019.
